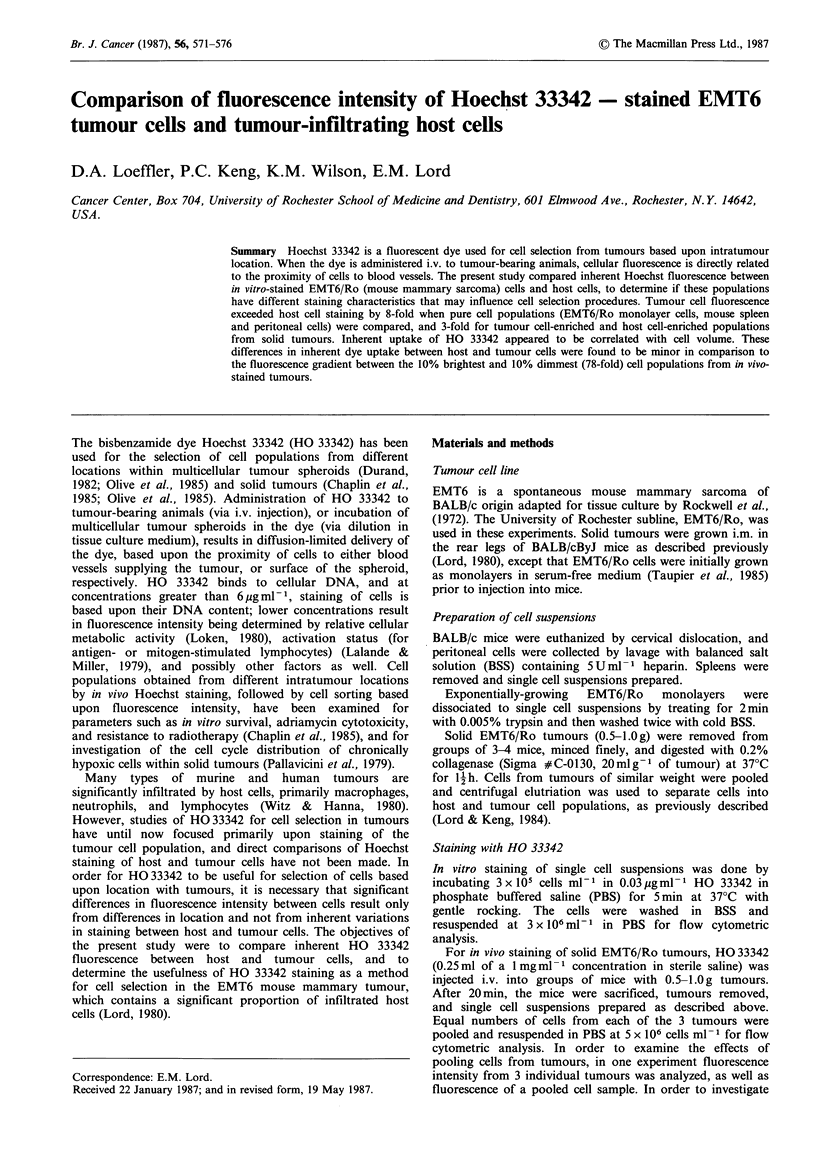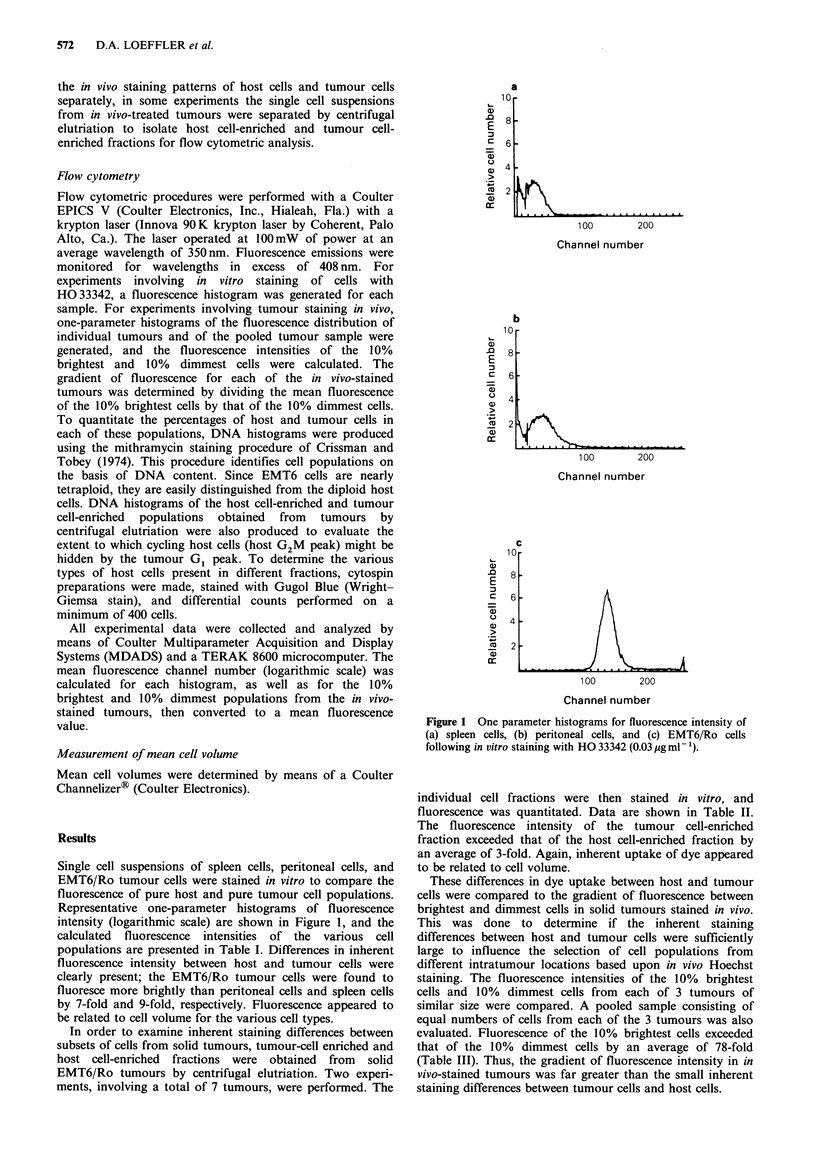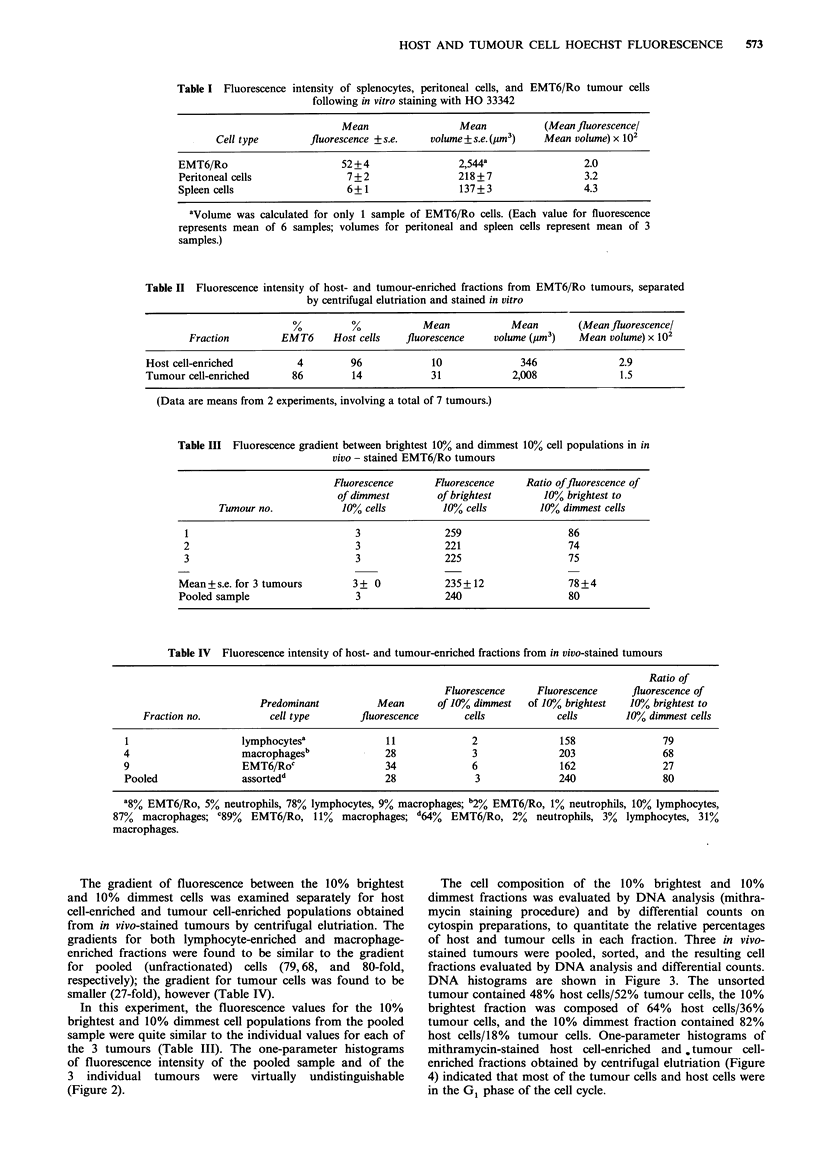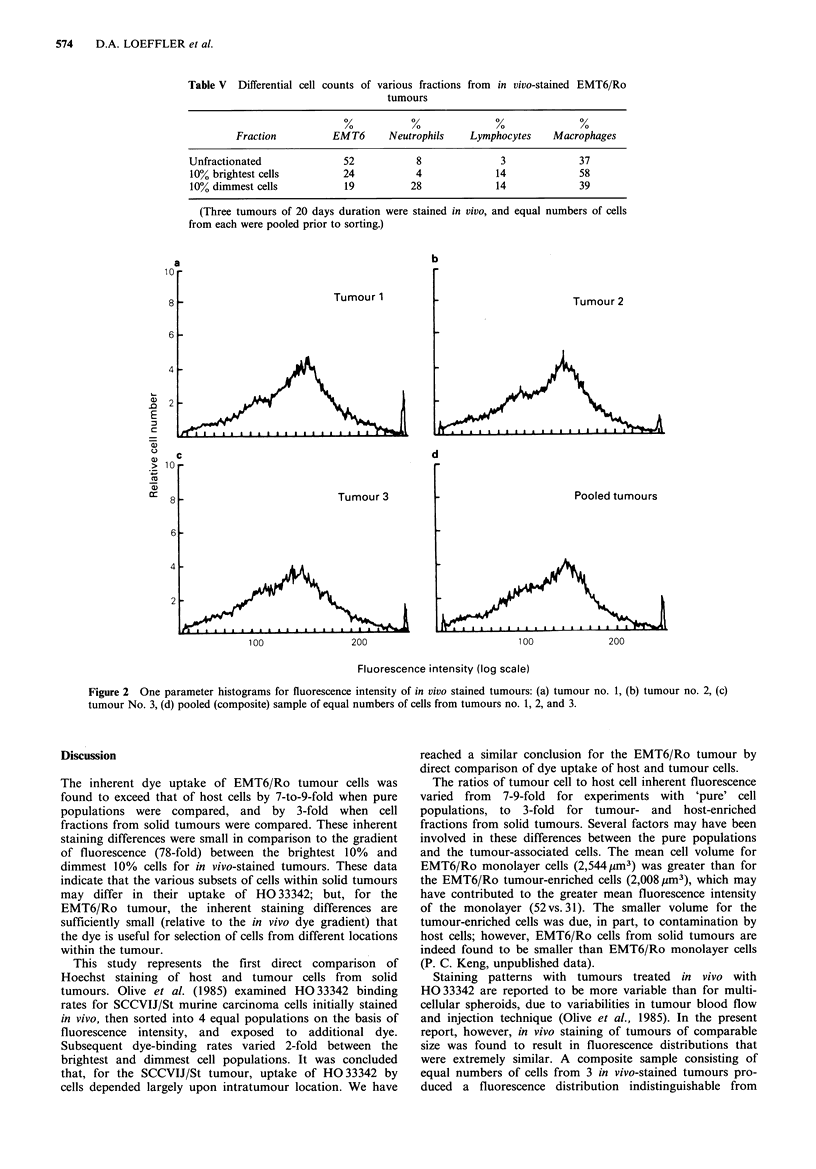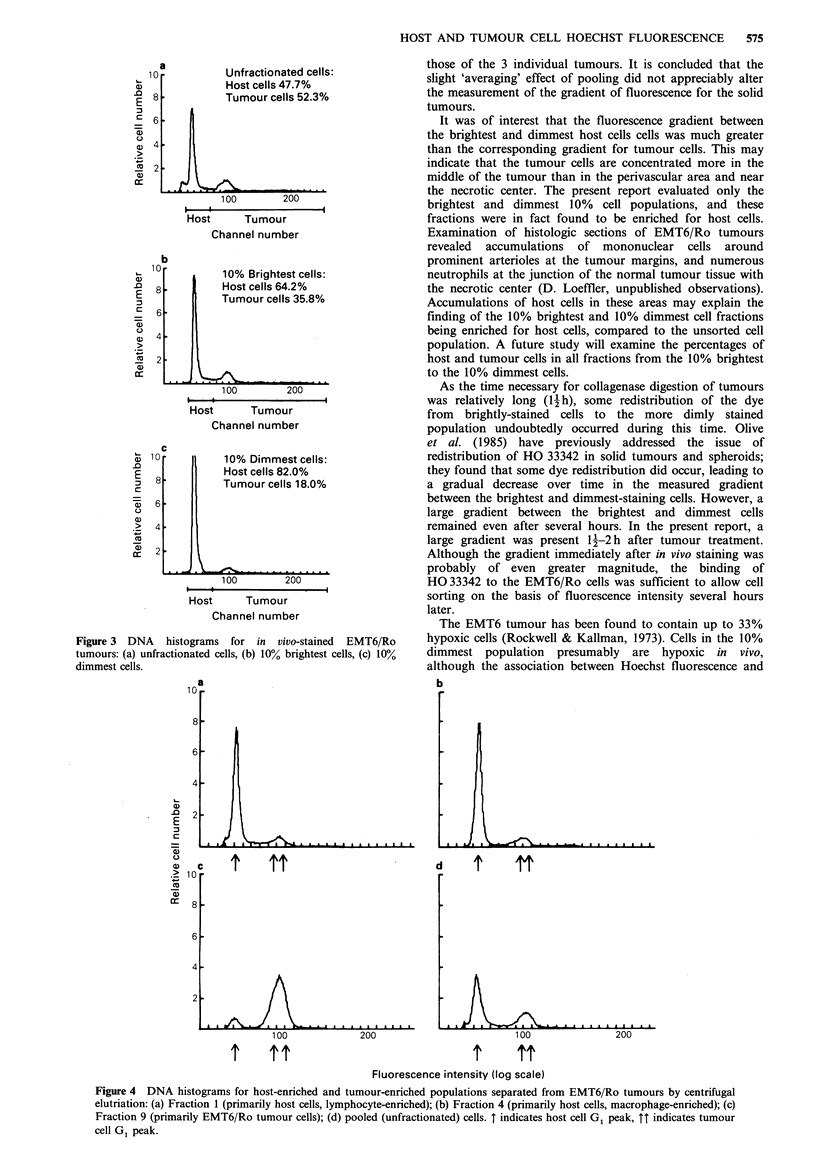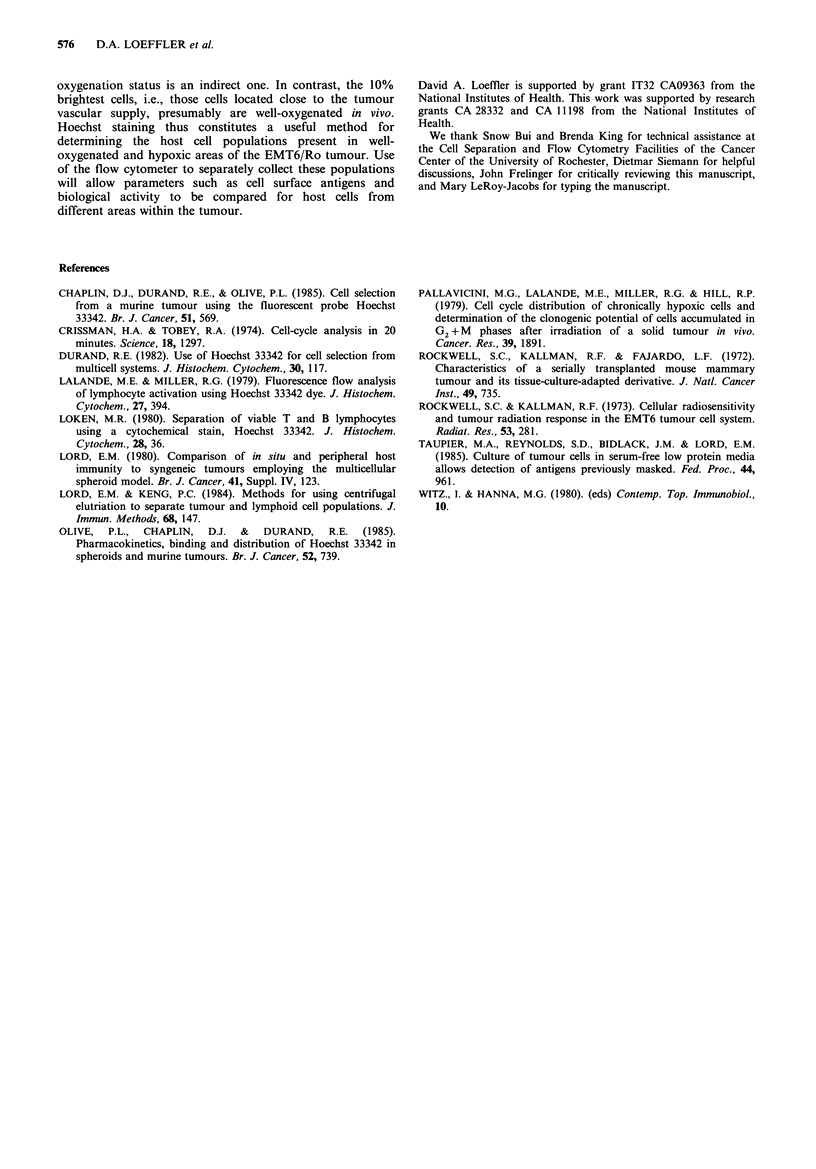# Comparison of fluorescence intensity of Hoechst 33342-stained EMT6 tumour cells and tumour-infiltrating host cells.

**DOI:** 10.1038/bjc.1987.243

**Published:** 1987-11

**Authors:** D. A. Loeffler, P. C. Keng, K. M. Wilson, E. M. Lord

**Affiliations:** Cancer Center, University of Rochester School of Medicine and Dentistry, N.Y. 14642.

## Abstract

Hoechst 33342 is a fluorescent dye used for cell selection from tumours based upon intratumour location. When the dye is administered i.v. to tumour-bearing animals, cellular fluorescence is directly related to the proximity of cells to blood vessels. The present study compared inherent Hoechst fluorescence between in vitro-stained EMT6/Ro (mouse mammary sarcoma) cells and host cells, to determine if these populations have different staining characteristics that may influence cell selection procedures. Tumour cell fluorescence exceeded host cell staining by 8-fold when pure cell populations (EMT6/Ro monolayer cells, mouse spleen and peritoneal cells) were compared, and 3-fold for tumour cell-enriched and host cell-enriched populations from solid tumours. Inherent uptake of HO 33342 appeared to be correlated with cell volume. These differences in inherent dye uptake between host and tumour cells were found to be minor in comparison to the fluorescence gradient between the 10% brightest and 10% dimmest (78-fold) cell populations from in vivo-stained tumours.


Br. J. Cancer (1987), 56, 571–576 © The Macmillan Press Ltd., 1987

### Comparison of fluorescence intensity of Hoechst 33342 — stained EMT6 tumour cells and tumour-infiltrating host cells

D.A. Loeffler, P.C. Keng, K.M. Wilson, E.M. Lord

*Cancer Center, Box 704, University of Rochester School of Medicine and Dentistry, 601 Elmwood Ave., Rochester, N.Y. 14642, USA.*

**Summary** Hoechst 33342 is a fluorescent dye used for cell selection from tumours based upon intratumour location. When the dye is administered i.v. to tumour-bearing animals, cellular fluorescence is directly related to the proximity of cells to blood vessels. The present study compared inherent Hoechst fluorescence between *in vitro*-stained EMT6/Ro (mouse mammary sarcoma) cells and host cells, to determine if these populations have different staining characteristics that may influence cell selection procedures. Tumour cell fluorescence exceeded host cell staining by 8-fold when pure cell populations (EMT6/Ro monolayer cells, mouse spleen and peritoneal cells) were compared, and 3-fold for tumour cell-enriched and host cell-enriched populations from solid tumours. Inherent uptake of HO 33342 appeared to be correlated with cell volume. These differences in inherent dye uptake between host and tumour cells were found to be minor in comparison to the fluorescence gradient between the 10% brightest and 10% dimmest (78-fold) cell populations from *in vivo*-stained tumours.

The bisbenzamide dye Hoechst 33342 (HO 33342) has been used for the selection of cell populations from different locations within multicellular tumour spheroids (Durand, 1982; Olive *et al.*, 1985) and solid tumours (Chaplin *et al.*, 1985; Olive *et al.*, 1985). Administration of HO 33342 to tumour-bearing animals (via i.v. injection), or incubation of multicellular tumour spheroids in the dye (via dilution in tissue culture medium), results in diffusion-limited delivery of the dye, based upon the proximity of cells to either blood vessels supplying the tumour, or surface of the spheroid, respectively. HO 33342 binds to cellular DNA, and at concentrations greater than $6\mu g\,ml^{-1}$, staining of cells is based upon their DNA content; lower concentrations result in fluorescence intensity being determined by relative cellular metabolic activity (Loken, 1980), activation status (for antigen- or mitogen-stimulated lymphocytes) (Lalande & Miller, 1979), and possibly other factors as well. Cell populations obtained from different intratumour locations by *in vivo* Hoechst staining, followed by cell sorting based upon fluorescence intensity, have been examined for parameters such as *in vitro* survival, adriamycin cytotoxicity, and resistance to radiotherapy (Chaplin *et al.*, 1985), and for investigation of the cell cycle distribution of chronically hypoxic cells within solid tumours (Pallavicini *et al.*, 1979).

Many types of murine and human tumours are significantly infiltrated by host cells, primarily macrophages, neutrophils, and lymphocytes (Witz & Hanna, 1980). However, studies of HO 33342 for cell selection in tumours have until now focused primarily upon staining of the tumour cell population, and direct comparisons of Hoechst staining of host and tumour cells have not been made. In order for HO 33342 to be useful for selection of cells based upon location with tumours, it is necessary that significant differences in fluorescence intensity between cells result only from differences in location and not from inherent variations in staining between host and tumour cells. The objectives of the present study were to compare inherent HO 33342 fluorescence between host and tumour cells, and to determine the usefulness of HO 33342 staining as a method for cell selection in the EMT6 mouse mammary tumour, which contains a significant proportion of infiltrated host cells (Lord, 1980).

Correspondence: E.M. Lord.
Received 22 January 1987; and in revised form, 19 May 1987.

### Materials and methods

### *Tumour cell line*

EMT6 is a spontaneous mouse mammary sarcoma of BALB/c origin adapted for tissue culture by Rockwell *et al.*, (1972). The University of Rochester subline, EMT6/Ro, was used in these experiments. Solid tumours were grown i.m. in the rear legs of BALB/cByJ mice as described previously (Lord, 1980), except that EMT6/Ro cells were initially grown as monolayers in serum-free medium (Taupier *et al.*, 1985) prior to injection into mice.

### *Preparation of cell suspensions*

BALB/c mice were euthanized by cervical dislocation, and peritoneal cells were collected by lavage with balanced salt solution (BSS) containing $5\,U\,ml^{-1}$ heparin. Spleens were removed and single cell suspensions prepared.

Exponentially-growing EMT6/Ro monolayers were dissociated to single cell suspensions by treating for 2 min with 0.005% trypsin and then washed twice with cold BSS.

Solid EMT6/Ro tumours (0.5–1.0 g) were removed from groups of 3–4 mice, minced finely, and digested with 0.2% collagenase (Sigma #C-0130, $20\,ml\,g^{-1}$ of tumour) at 37°C for $1\frac{1}{2}$ h. Cells from tumours of similar weight were pooled and centrifugal elutriation was used to separate cells into host and tumour cell populations, as previously described (Lord & Keng, 1984).

### *Staining with HO 33342*

*In vitro* staining of single cell suspensions was done by incubating $3\times10^5$ cells $ml^{-1}$ in $0.03\,\mu g\,ml^{-1}$ HO 33342 in phosphate buffered saline (PBS) for 5 min at 37°C with gentle rocking. The cells were washed in BSS and resuspended at $3\times10^6\,ml^{-1}$ in PBS for flow cytometric analysis.

For *in vivo* staining of solid EMT6/Ro tumours, HO 33342 (0.25 ml of a $1\,mg\,ml^{-1}$ concentration in sterile saline) was injected i.v. into groups of mice with 0.5–1.0 g tumours. After 20 min, the mice were sacrificed, tumours removed, and single cell suspensions prepared as described above. Equal numbers of cells from each of the 3 tumours were pooled and resuspended in PBS at $5\times10^6$ cells $ml^{-1}$ for flow cytometric analysis. In order to examine the effects of pooling cells from tumours, in one experiment fluorescence intensity from 3 individual tumours was analyzed, as well as fluorescence of a pooled cell sample. In order to investigate the *in vivo* staining patterns of host cells and tumour cells separately, in some experiments the single cell suspensions from *in vivo*-treated tumours were separated by centrifugal elutriation to isolate host cell-enriched and tumour cell-enriched fractions for flow cytometric analysis.

### *Flow cytometry*

Flow cytometric procedures were performed with a Coulter EPICS V (Coulter Electronics, Inc., Hialeah, Fla.) with a krypton laser (Innova 90 K krypton laser by Coherent, Palo Alto, Ca.). The laser operated at 100 mW of power at an average wavelength of 350 nm. Fluorescence emissions were monitored for wavelengths in excess of 408 nm. For experiments involving *in vitro* staining of cells with HO 33342, a fluorescence histogram was generated for each sample. For experiments involving tumour staining *in vivo*, one-parameter histograms of the fluorescence distribution of individual tumours and of the pooled tumour sample were generated, and the fluorescence intensities of the 10% brightest and 10% dimmest cells were calculated. The gradient of fluorescence for each of the *in vivo*-stained tumours was determined by dividing the mean fluorescence of the 10% brightest cells by that of the 10% dimmest cells. To quantitate the percentages of host and tumour cells in each of these populations, DNA histograms were produced using the mithramycin staining procedure of Crissman and Tobey (1974). This procedure identifies cell populations on the basis of DNA content. Since EMT6 cells are nearly tetraploid, they are easily distinguished from the diploid host cells. DNA histograms of the host cell-enriched and tumour cell-enriched populations obtained from tumours by centrifugal elutriation were also produced to evaluate the extent to which cycling host cells (host $G_2M$ peak) might be hidden by the tumour $G_1$ peak. To determine the various types of host cells present in different fractions, cytospin preparations were made, stained with Gugol Blue (Wright–Giemsa stain), and differential counts performed on a minimum of 400 cells.

All experimental data were collected and analyzed by means of Coulter Multiparameter Acquisition and Display Systems (MDADS) and a TERAK 8600 microcomputer. The mean fluorescence channel number (logarithmic scale) was calculated for each histogram, as well as for the 10% brightest and 10% dimmest populations from the *in vivo*-stained tumours, then converted to a mean fluorescence value.

### *Measurement of mean cell volume*

Mean cell volumes were determined by means of a Coulter Channelizer® (Coulter Electronics).

### Results

Single cell suspensions of spleen cells, peritoneal cells, and EMT6/Ro tumour cells were stained *in vitro* to compare the fluorescence of pure host and pure tumour cell populations. Representative one-parameter histograms of fluorescence intensity (logarithmic scale) are shown in Figure 1, and the calculated fluorescence intensities of the various cell populations are presented in Table I. Differences in inherent fluorescence intensity between host and tumour cells were clearly present; the EMT6/Ro tumour cells were found to fluoresce more brightly than peritoneal cells and spleen cells by 7-fold and 9-fold, respectively. Fluorescence appeared to be related to cell volume for the various cell types.

In order to examine inherent staining differences between subsets of cells from solid tumours, tumour-cell enriched and host cell-enriched fractions were obtained from solid EMT6/Ro tumours by centrifugal elutriation. Two experiments, involving a total of 7 tumours, were performed. The

**Figure 1** One parameter histograms for fluorescence intensity of (a) spleen cells, (b) peritoneal cells, and (c) EMT6/Ro cells following *in vitro* staining with HO 33342 (0.03 $\mu g\,ml^{-1}$).

individual cell fractions were then stained *in vitro*, and fluorescence was quantitated. Data are shown in Table II. The fluorescence intensity of the tumour cell-enriched fraction exceeded that of the host cell-enriched fraction by an average of 3-fold. Again, inherent uptake of dye appeared to be related to cell volume.

These differences in dye uptake between host and tumour cells were compared to the gradient of fluorescence between brightest and dimmest cells in solid tumours stained *in vivo*. This was done to determine if the inherent staining differences between host and tumour cells were sufficiently large to influence the selection of cell populations from different intratumour locations based upon *in vivo* Hoechst staining. The fluorescence intensities of the 10% brightest cells and 10% dimmest cells from each of 3 tumours of similar size were compared. A pooled sample consisting of equal numbers of cells from each of the 3 tumours was also evaluated. Fluorescence of the 10% brightest cells exceeded that of the 10% dimmest cells by an average of 78-fold (Table III). Thus, the gradient of fluorescence intensity in *in vivo*-stained tumours was far greater than the small inherent staining differences between tumour cells and host cells.

**Table I** Fluorescence intensity of splenocytes, peritoneal cells, and EMT6/Ro tumour cells following *in vitro* staining with HO 33342

| *Cell type* | *Mean fluorescence* ±*s.e.* | *Mean volume*±*s.e.* ($\mu m^3$) | (*Mean fluorescence/ Mean volume*) × $10^2$ |
|---|---|---|---|
| EMT6/Ro | 52±4 | 2,544[a] | 2.0 |
| Peritoneal cells | 7±2 | 218±7 | 3.2 |
| Spleen cells | 6±1 | 137±3 | 4.3 |

[a]Volume was calculated for only 1 sample of EMT6/Ro cells. (Each value for fluorescence represents mean of 6 samples; volumes for peritoneal and spleen cells represent mean of 3 samples.)

**Table II** Fluorescence intensity of host- and tumour-enriched fractions from EMT6/Ro tumours, separated by centrifugal elutriation and stained *in vitro*

| *Fraction* | % *EMT6* | % *Host cells* | *Mean fluorescence* | *Mean volume* ($\mu m^3$) | (*Mean fluorescence/ Mean volume*) × $10^2$ |
|---|---|---|---|---|---|
| Host cell-enriched | 4 | 96 | 10 | 346 | 2.9 |
| Tumour cell-enriched | 86 | 14 | 31 | 2,008 | 1.5 |

(Data are means from 2 experiments, involving a total of 7 tumours.)

**Table III** Fluorescence gradient between brightest 10% and dimmest 10% cell populations in *in vivo* – stained EMT6/Ro tumours

| *Tumour no.* | *Fluorescence of dimmest 10% cells* | *Fluorescence of brightest 10% cells* | *Ratio of fluorescence of 10% brightest to 10% dimmest cells* |
|---|---|---|---|
| 1 | 3 | 259 | 86 |
| 2 | 3 | 221 | 74 |
| 3 | 3 | 225 | 75 |
| — | — | — | — |
| Mean±s.e. for 3 tumours | 3± 0 | 235±12 | 78±4 |
| Pooled sample | 3 | 240 | 80 |

**Table IV** Fluorescence intensity of host- and tumour-enriched fractions from *in vivo*-stained tumours

| *Fraction no.* | *Predominant cell type* | *Mean fluorescence* | *Fluorescence of 10% dimmest cells* | *Fluorescence of 10% brightest cells* | *Ratio of fluorescence of 10% brightest to 10% dimmest cells* |
|---|---|---|---|---|---|
| 1 | lymphocytes[a] | 11 | 2 | 158 | 79 |
| 4 | macrophages[b] | 28 | 3 | 203 | 68 |
| 9 | EMT6/Ro[c] | 34 | 6 | 162 | 27 |
| Pooled | assorted[d] | 28 | 3 | 240 | 80 |

[a]8% EMT6/Ro, 5% neutrophils, 78% lymphocytes, 9% macrophages; [b]2% EMT6/Ro, 1% neutrophils, 10% lymphocytes, 87% macrophages; [c]89% EMT6/Ro, 11% macrophages; [d]64% EMT6/Ro, 2% neutrophils, 3% lymphocytes, 31% macrophages.

The gradient of fluorescence between the 10% brightest and 10% dimmest cells was examined separately for host cell-enriched and tumour cell-enriched populations obtained from *in vivo*-stained tumours by centrifugal elutriation. The gradients for both lymphocyte-enriched and macrophage-enriched fractions were found to be similar to the gradient for pooled (unfractionated) cells (79, 68, and 80-fold, respectively); the gradient for tumour cells was found to be smaller (27-fold), however (Table IV).

In this experiment, the fluorescence values for the 10% brightest and 10% dimmest cell populations from the pooled sample were quite similar to the individual values for each of the 3 tumours (Table III). The one-parameter histograms of fluorescence intensity of the pooled sample and of the 3 individual tumours were virtually undistinguishable (Figure 2).

The cell composition of the 10% brightest and 10% dimmest fractions was evaluated by DNA analysis (mithramycin staining procedure) and by differential counts on cytospin preparations, to quantitate the relative percentages of host and tumour cells in each fraction. Three *in vivo*-stained tumours were pooled, sorted, and the resulting cell fractions evaluated by DNA analysis and differential counts. DNA histograms are shown in Figure 3. The unsorted tumour contained 48% host cells/52% tumour cells, the 10% brightest fraction was composed of 64% host cells/36% tumour cells, and the 10% dimmest fraction contained 82% host cells/18% tumour cells. One-parameter histograms of mithramycin-stained host cell-enriched and tumour cell-enriched fractions obtained by centrifugal elutriation (Figure 4) indicated that most of the tumour cells and host cells were in the $G_1$ phase of the cell cycle.

**Table V** Differential cell counts of various fractions from *in vivo*-stained EMT6/Ro tumours

| Fraction | % EMT6 | % Neutrophils | % Lymphocytes | % Macrophages |
|---|---|---|---|---|
| Unfractionated | 52 | 8 | 3 | 37 |
| 10% brightest cells | 24 | 4 | 14 | 58 |
| 10% dimmest cells | 19 | 28 | 14 | 39 |

(Three tumours of 20 days duration were stained *in vivo*, and equal numbers of cells from each were pooled prior to sorting.)

**Figure 2** One parameter histograms for fluorescence intensity of *in vivo* stained tumours: (a) tumour no. 1, (b) tumour no. 2, (c) tumour No. 3, (d) pooled (composite) sample of equal numbers of cells from tumours no. 1, 2, and 3.

### Discussion

The inherent dye uptake of EMT6/Ro tumour cells was found to exceed that of host cells by 7-to-9-fold when pure populations were compared, and by 3-fold when cell fractions from solid tumours were compared. These inherent staining differences were small in comparison to the gradient of fluorescence (78-fold) between the brightest 10% and dimmest 10% cells for *in vivo*-stained tumours. These data indicate that the various subsets of cells within solid tumours may differ in their uptake of HO 33342; but, for the EMT6/Ro tumour, the inherent staining differences are sufficiently small (relative to the *in vivo* dye gradient) that the dye is useful for selection of cells from different locations within the tumour.

This study represents the first direct comparison of Hoechst staining of host and tumour cells from solid tumours. Olive *et al.* (1985) examined HO 33342 binding rates for SCCVIJ/St murine carcinoma cells initially stained *in vivo*, then sorted into 4 equal populations on the basis of fluorescence intensity, and exposed to additional dye. Subsequent dye-binding rates varied 2-fold between the brightest and dimmest cell populations. It was concluded that, for the SCCVIJ/St tumour, uptake of HO 33342 by cells depended largely upon intratumour location. We have reached a similar conclusion for the EMT6/Ro tumour by direct comparison of dye uptake of host and tumour cells.

The ratios of tumour cell to host cell inherent fluorescence varied from 7-9-fold for experiments with 'pure' cell populations, to 3-fold for tumour- and host-enriched fractions from solid tumours. Several factors may have been involved in these differences between the pure populations and the tumour-associated cells. The mean cell volume for EMT6/Ro monolayer cells (2,544 $\mu m^3$) was greater than for the EMT6/Ro tumour-enriched cells (2,008 $\mu m^3$), which may have contributed to the greater mean fluorescence intensity of the monolayer (52 vs. 31). The smaller volume for the tumour-enriched cells was due, in part, to contamination by host cells; however, EMT6/Ro cells from solid tumours are indeed found to be smaller than EMT6/Ro monolayer cells (P. C. Keng, unpublished data).

Staining patterns with tumours treated *in vivo* with HO 33342 are reported to be more variable than for multicellular spheroids, due to variabilities in tumour blood flow and injection technique (Olive *et al.*, 1985). In the present report, however, *in vivo* staining of tumours of comparable size was found to result in fluorescence distributions that were extremely similar. A composite sample consisting of equal numbers of cells from 3 *in vivo*-stained tumours produced a fluorescence distribution indistinguishable from

**Figure 3** DNA histograms for *in vivo*-stained EMT6/Ro tumours: (a) unfractionated cells, (b) 10% brightest cells, (c) 10% dimmest cells.

those of the 3 individual tumours. It is concluded that the slight 'averaging' effect of pooling did not appreciably alter the measurement of the gradient of fluorescence for the solid tumours.

It was of interest that the fluorescence gradient between the brightest and dimmest host cells cells was much greater than the corresponding gradient for tumour cells. This may indicate that the tumour cells are concentrated more in the middle of the tumour than in the perivascular area and near the necrotic center. The present report evaluated only the brightest and dimmest 10% cell populations, and these fractions were in fact found to be enriched for host cells. Examination of histologic sections of EMT6/Ro tumours revealed accumulations of mononuclear cells around prominent arterioles at the tumour margins, and numerous neutrophils at the junction of the normal tumour tissue with the necrotic center (D. Loeffler, unpublished observations). Accumulations of host cells in these areas may explain the finding of the 10% brightest and 10% dimmest cell fractions being enriched for host cells, compared to the unsorted cell population. A future study will examine the percentages of host and tumour cells in all fractions from the 10% brightest to the 10% dimmest cells.

As the time necessary for collagenase digestion of tumours was relatively long ($1\frac{1}{2}$ h), some redistribution of the dye from brightly-stained cells to the more dimly stained population undoubtedly occurred during this time. Olive *et al.* (1985) have previously addressed the issue of redistribution of HO 33342 in solid tumours and spheroids; they found that some dye redistribution did occur, leading to a gradual decrease over time in the measured gradient between the brightest and dimmest-staining cells. However, a large gradient between the brightest and dimmest cells remained even after several hours. In the present report, a large gradient was present $1\frac{1}{2}$–2 h after tumour treatment. Although the gradient immediately after *in vivo* staining was probably of even greater magnitude, the binding of HO 33342 to the EMT6/Ro cells was sufficient to allow cell sorting on the basis of fluorescence intensity several hours later.

The EMT6 tumour has been found to contain up to 33% hypoxic cells (Rockwell & Kallman, 1973). Cells in the 10% dimmest population presumably are hypoxic *in vivo*, although the association between Hoechst fluorescence and

**Figure 4** DNA histograms for host-enriched and tumour-enriched populations separated from EMT6/Ro tumours by centrifugal elutriation: (a) Fraction 1 (primarily host cells, lymphocyte-enriched); (b) Fraction 4 (primarily host cells, macrophage-enriched); (c) Fraction 9 (primarily EMT6/Ro tumour cells); (d) pooled (unfractionated) cells. ↑ indicates host cell $G_1$ peak, ↑↑ indicates tumour cell $G_1$ peak.

oxygenation status is an indirect one. In contrast, the 10% brightest cells, i.e., those cells located close to the tumour vascular supply, presumably are well-oxygenated *in vivo*. Hoechst staining thus constitutes a useful method for determining the host cell populations present in well-oxygenated and hypoxic areas of the EMT6/Ro tumour. Use of the flow cytometer to separately collect these populations will allow parameters such as cell surface antigens and biological activity to be compared for host cells from different areas within the tumour.

David A. Loeffler is supported by grant IT32 CA09363 from the National Institutes of Health. This work was supported by research grants CA 28332 and CA 11198 from the National Institutes of Health.

We thank Snow Bui and Brenda King for technical assistance at the Cell Separation and Flow Cytometry Facilities of the Cancer Center of the University of Rochester, Dietmar Siemann for helpful discussions, John Frelinger for critically reviewing this manuscript, and Mary LeRoy-Jacobs for typing the manuscript.

### References

CHAPLIN, D.J., DURAND, R.E., & OLIVE, P.L. (1985). Cell selection from a murine tumour using the fluorescent probe Hoechst 33342. *Br. J. Cancer*, **51**, 569.

CRISSMAN, H.A. & TOBEY, R.A. (1974). Cell-cycle analysis in 20 minutes. *Science*, **18**, 1297.

DURAND, R.E. (1982). Use of Hoechst 33342 for cell selection from multicell systems. *J. Histochem. Cytochem.*, **30**, 117.

LALANDE, M.E. & MILLER, R.G. (1979). Fluorescence flow analysis of lymphocyte activation using Hoechst 33342 dye. *J. Histochem. Cytochem.*, **27**, 394.

LOKEN, M.R. (1980). Separation of viable T and B lymphocytes using a cytochemical stain, Hoechst 33342. *J. Histochem. Cytochem.*, **28**, 36.

LORD, E.M. (1980). Comparison of *in situ* and peripheral host immunity to syngeneic tumours employing the multicellular spheroid model. *Br. J. Cancer*, **41**, Suppl. IV, 123.

LORD, E.M. & KENG, P.C. (1984). Methods for using centrifugal elutriation to separate tumour and lymphoid cell populations. *J. Immun. Methods*, **68**, 147.

OLIVE, P.L., CHAPLIN, D.J. & DURAND, R.E. (1985). Pharmacokinetics, binding and distribution of Hoechst 33342 in spheroids and murine tumours. *Br. J. Cancer*, **52**, 739.

PALLAVICINI, M.G., LALANDE, M.E., MILLER, R.G. & HILL, R.P. (1979). Cell cycle distribution of chronically hypoxic cells and determination of the clonogenic potential of cells accumulated in $G_2+M$ phases after irradiation of a solid tumour *in vivo*. *Cancer. Res.*, **39**, 1891.

ROCKWELL, S.C., KALLMAN, R.F. & FAJARDO, L.F. (1972). Characteristics of a serially transplanted mouse mammary tumour and its tissue-culture-adapted derivative. *J. Natl. Cancer Inst.*, **49**, 735.

ROCKWELL, S.C. & KALLMAN, R.F. (1973). Cellular radiosensitivity and tumour radiation response in the EMT6 tumour cell system. *Radiat. Res.*, **53**, 281.

TAUPIER, M.A., REYNOLDS, S.D., BIDLACK, J.M. & LORD, E.M. (1985). Culture of tumour cells in serum-free low protein media allows detection of antigens previously masked. *Fed. Proc.*, **44**, 961.

WITZ., I. & HANNA, M.G. (1980). (eds) *Contemp. Top. Immunobiol.*, **10**.